# Genome Sequence of Pyrethroid-Degrading Bacterium *Rhodopseudomonas palustris* Strain JSC-3b

**DOI:** 10.1128/genomeA.01228-13

**Published:** 2014-01-23

**Authors:** Songbai Zhang, Xiangwen Luo, Ju’e Cheng, Jing Peng, Deyong Zhang, Yong Liu

**Affiliations:** a Key Laboratory of Pest Management of Horticultural Crop of Hunan Province, Hunan Plant Protection Institute, Changsha, China; b Branch of Longping, Graduate College, Central South University, Changsha, China

## Abstract

*Rhodopseudomonas palustris* strain JSC-3b is a facultative, thermophilic bacterium, which was isolated from water in a canal adjacent to a vegetable field. Strain JSC-3b biodegrades several varieties of pyrethroid residues effectively through cometabolic pathways. Here, we present the genome sequence of this biodegrader.

## GENOME ANNOUNCEMENT

Pyrethroid pesticides, a large class of synthetic compounds based on the structure of pyrethrin, have been extensively utilized for more than 30 years for controlling agriculture, forestry, and indoor pests (1). For quite a long time in the past, pyrethroids were believed to have low toxicity to the environment and were considered an ideal replacement for some varieties of highly toxic organochlorine and organophosphorus pesticides (2). However, recent studies have verified that pyrethroids are extremely toxic to aquatic invertebrates (3). In addition, pyrethroids have been demonstrated by animal experiments to induce neurotoxicity, reproductive toxicity, cytotoxicity, teratogenicity, and carcinogenicity (4–7). Some pyrethroids have been classified as potential human carcinogens by the U.S. Environmental Protection Agency (8). Thus, the negative effects of pyrethroids to public health and environmental safety are increasing.

Pyrethroid residues can be cleaned up by abiotic or biotic modes in natural niches. Of the biotic modes, microbial decomposition has been verified as a key function for degrading pyrethroid residuals (9). Although some degrading microorganisms and their degrading enzymes and genes have been screened and studied (10–12), we are still far from understanding the explicit mechanisms of pyrethroid biodegradation.

The draft genomic data of strain JSC-3b were sequenced by an Illumina HighSeq 2000 sequencer using two paired-end libraries. There were 8,803,720 reads and 4,549,174 reads generated from 6-kb and 800-bp libraries, respectively. After removal of quality reads, the clean data consisted of 585 Mb and 301 Mb, respectively. The clean data were further assembled with SOAPdenovo (http://soap.genomics.org.cn/soapdenovo.html), version 2.04 (13). The clean data of strain JSC-3b contain 43 contigs covering a total of 4,065,668 bp. The maximum and minimum lengths of contigs were 725,135 bp and 391 bp, respectively, and the *N*_50_ contig size was 204,823 bp. The genome size of strain JSC-3b was approximately 4,068,467 bp, with an overall estimated GC content of 64.22%. The genome sequence was then *de novo* annotated with different genomic software tools. Open reading frames (ORFs) were predicted using Glimmer (14). Tandem repeats were identified by Tandem Repeat Finder (version 4.04) (15). rRNAs and tRNAs were identified by RNAmmer (16) and tRNAscan-SE 1.23 (17). The total genome is composed of 3,799 coding sequences, and the total length of predicted genes was 3,444,753 bp, which makes up 84.67% of the whole-genome sequence. There are 351 tandem repeat sequences, and the total length of the tandem repeat sequence was 24,289 bp, which is approximately 0.60% of the whole-genome sequence. The numbers of minisatellite DNAs and microsatellite DNAs were 258 and 8, respectively. The genome also contains 8 copies of tRNA and 10 copies of rRNA.

This study reports the *R. palustris* strain JSC-3b genome sequence for the first time. To the best of our knowledge, this is the first report of genomic data of *R. palustris*, which has the capacity to effectively degrade pyrethroids. This genome sequence will facilitate insight into pyrethroid tolerance and degradation mechanisms.

### Nucleotide sequence accession number.

The draft genome sequence of *R. palustris* strain JSC-3b has been deposited in DDBJ/EMBL/GenBank under accession number AYSU00000000. The version presented in this paper is the first version.
